# Ageing-associated changes in the human DNA methylome: genomic locations and effects on gene expression

**DOI:** 10.1186/s12864-015-1381-z

**Published:** 2015-03-14

**Authors:** Saara Marttila, Laura Kananen, Sergei Häyrynen, Juulia Jylhävä, Tapio Nevalainen, Antti Hervonen, Marja Jylhä, Matti Nykter, Mikko Hurme

**Affiliations:** Department of Microbiology and Immunology, School of Medicine, University of Tampere, Tampere, Finland; Gerontology Research Center, Tampere, Finland; Institute of Biosciences and Medical Technology, University of Tampere, Tampere, Finland; School of Health Sciences, University of Tampere, Tampere, Finland; Fimlab Laboratories, Tampere, Finland

**Keywords:** Epigenetics, Methylome, DNA methylation, Ageing, PBMCs, Gene expression, Molecular ageing, Hypermethylation, Hypomethylation

## Abstract

**Background:**

Changes in DNA methylation are among the mechanisms contributing to the ageing process. We sought to identify ageing-associated DNA methylation changes at single-CpG-site resolution in blood leukocytes and to ensure that the observed changes were not due to differences in the proportions of leukocytes. The association between DNA methylation changes and gene expression levels was also investigated in the same individuals.

**Results:**

We identified 8540 high-confidence ageing-associated CpG sites, 46% of which were hypermethylated in nonagenarians. The hypermethylation-associated genes belonged to a common category: they were predicted to be regulated by a common group of transcription factors and were enriched in a related set of GO terms and canonical pathways. Conversely, for the hypomethylation-associated genes only a limited set of GO terms and canonical pathways were identified. Among the 8540 CpG sites associated with ageing, methylation level of 377 sites was also associated with gene expression levels. These genes were enriched in GO terms and canonical pathways associated with immune system functions, particularly phagocytosis.

**Conclusions:**

We find that certain ageing-associated immune-system impairments may be mediated via changes in DNA methylation. The results also imply that ageing-associated hypo- and hypermethylation are distinct processes: hypermethylation could be caused by programmed changes, whereas hypomethylation could be the result of environmental and stochastic processes.

**Electronic supplementary material:**

The online version of this article (doi:10.1186/s12864-015-1381-z) contains supplementary material, which is available to authorized users.

## Background

Ageing can be described as a functional decline that leads to a diminished ability to respond to stress, increased homeostatic instability and an increased risk of diseases such as cancer and inflammatory diseases. Ultimately, these changes lead to death [1]. The molecular basis of ageing is multifactorial, including changes in energy metabolism, alterations in DNA repair mechanisms, increased inflammation and changes in leukocyte proportions (changes in CD4+/CD8+ ratio, increase of costimulatory CD28 receptor-deficient T cells [2]). Consequently, several theories exist regarding the mechanisms underlying ageing. Whether the ageing process itself consists of the accumulation of molecular damage due to environmental and stochastic effects or is a truly programmed or pseudo-programmed process that stems from development remains to be determined, yet a process as complex as ageing most likely involves aspects of all these phenomena [3-5].

Ageing leads to both global and local changes in the DNA methylation profile. Global hypomethylation has been shown to occur across tissues, and promoter-specific hypermethylation has been demonstrated for various tissues and genes [6]. Several ageing-relates diseases, such as cancer, Alzheimer’s disease and type 2 diabetes, have also been shown to be associated with changes in DNA methylation [7]. The role of epigenetics in ageing-associated processes could be significant, as genetics appears to explain only a small portion of the observed variation in lifespan and healthspan [8]. As the epigenome is modified throughout life by varying environmental conditions, the accumulated effects of these changes could be most prominent in the aged population.

DNA methylation was suggested to control the activity of genes as early as 1975 [9,10] and has since been demonstrated to control the expression of single genes and the silencing of large sections of chromatin. DNA methylation mainly occurs on CpG-dinucleotides, which form CpG islands containing above-average CpG content. These CpG islands overlap the transcription start sites (TSSs) of the majority of human genes, and the classical role of DNA methylation is transcriptional inhibition, with the methylation of TSSs preventing the initiation of transcription [11,12]. The role of methylation in the gene body is less clear; methylation does not appear to block transcriptional elongation but may actually enhance it, and methylation may have a role in alternative splicing. Furthermore, DNA methylation is required for the suppression of transposable elements [13]. DNA methylation controls gene expression by directly inhibiting the binding of transcription factors (TFs), by recruiting methyl-binding proteins that prevent TFs from binding to DNA [14], or by affecting the conformation of the surrounding chromatin [15].

The relationship between ageing and DNA methylation has been studied previously by measuring the DNA methylation level of repetitive elements (global DNA methylation [6]) as well as with Illumina Golden Gate array [16] and the Infinium HumanMethylation27 BeadChip (27 K array) [17-22]. These arrays included a severely biased set of CpGs located in known cancer-associated genes and CpGs located almost exclusively in CpG island promoter regions, respectively. The Illumina Infinium HumanMethylation450 BeadChip (450K array) offers an improvement in this area, as the probes span 99% of the RefSeq genes and are distributed more evenly across the genome, such as on the shores and shelves of CpG islands and in non-CpG islands (non-CGIs), as well as in gene bodies and untranslated regions (UTRs) [23-28]. However, the majority of previous studies did not take into consideration the prominent ageing-associated changes in the proportions of leukocytes, thereby introducing possible bias into the analyses [29].

In this study, our aim was to identify ageing-associated DNA methylation changes that are independent of changes in leukocyte proportions. We also examined gene expression data from the same individuals from whom the methylation data were obtained, and we were therefore able to explore the relationship between gene expression and DNA methylation in these elderly individuals.

## Results

### Ageing-associated DNA methylation changes

Our study population consisted of the Vitality 90+ study population: there was a total of 146 nonagenarians and 30 young controls, from whom we extracted peripheral blood mononuclear cells (PBMCs). The methylation data were produced with the 450K array, and the expression data were obtained with the Illumina HumanHT12v4 BeadChip. Our aim was to identify ageing-associated changes in the level of DNA methylation. Our approach was two-sided, as we sought to concentrate on CpG sites that showed a large enough difference in the level of methylation to have a plausible biological significance but also to ensure that the identified differences were not due to changes in the proportions of leukocyte populations.

**Figure 1:**
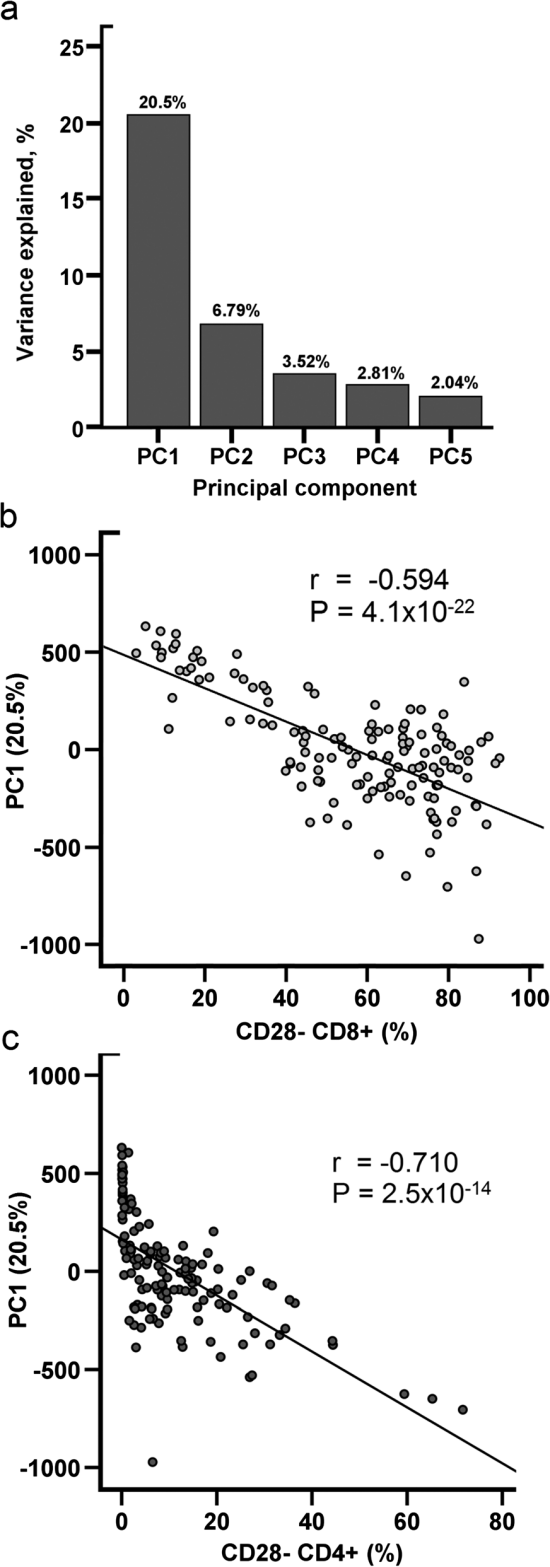
**The association of cell type proportions with DNA methylation.** The global DNA methylation was decomposed into a set of linearly independent principal component (PC) patterns. Components were used to examine the relationships between global DNA methylation and biological or non-biological covariates (e.g., gender, the batch effect and cell types). **(a)** The top 5 components (PC1-PC5) with the largest proportion of explained variance from the data. The percentages of explained variance are shown above the bars. **(b)** The association of the proportion of CD8 + CD28- cells with the first principal component (Spearman’s rank correlation coefficient -0.594 (p = 4.1e-22)) and **(c)** the association of the proportion of CD4 + CD28- cells with the first principal component (Spearman’s rank correlation coefficient -0.710 (p = 2.5e-14)).

The proportions of different leukocytes differed between the nonagenarians and young controls in our study population, as reported previously [30]. A principal component analysis (PCA) revealed that the first principal component accounted for 20.5% of the observed variation in methylation levels detected in our data (Figure 1). This component was strongly associated with leukocyte proportions, indicating that the analysis needs to be adjusted for the proportions of leukocytes.

First, we compared the methylation levels at individual CpGs in the nonagenarian group (n = 122) with those in the young control group (n = 21) using the Wilcoxon rank-sum test and identified 10083 CpG sites that were differentially methylated between these two groups (with a Benjamini-Hochberg-corrected p-value <0.05 and a difference between absolute M-value medians >1). Second, age group-associated methylation sites were identified with a beta regression model, with sex and different leukocyte populations (the ratio of CD4+ and CD8+ T cells and the proportions of CD4 + CD28-, CD8 + CD28- and CD14+ cells) as covariates. This method identified 45507 CpG sites for which age group was a significant covariate (Bonferroni-corrected p-value <0.05). The 10083 CpG sites identified via the group comparison were enriched at the top of the list of the 45507 ageing-associated CpGs. However, 1543 of the 10083 CpG sites showed no statistical significance in the regression analysis, indicating that the perceived difference in methylation was due to differences in leukocyte proportions rather than ageing *per se*. We now report the 8540 CpG sites, which exhibit a large, statistically significant difference in the level of methylation between the nonagenarians and young controls and remain significant after adjusting for differences in leukocyte populations in the regression analysis, as truly ageing-associated methylation changes (for a list of all ageing-associated CpGs, see Additional file 1). Sex chromosomes were excluded from the analysis.

**Figure 2:**
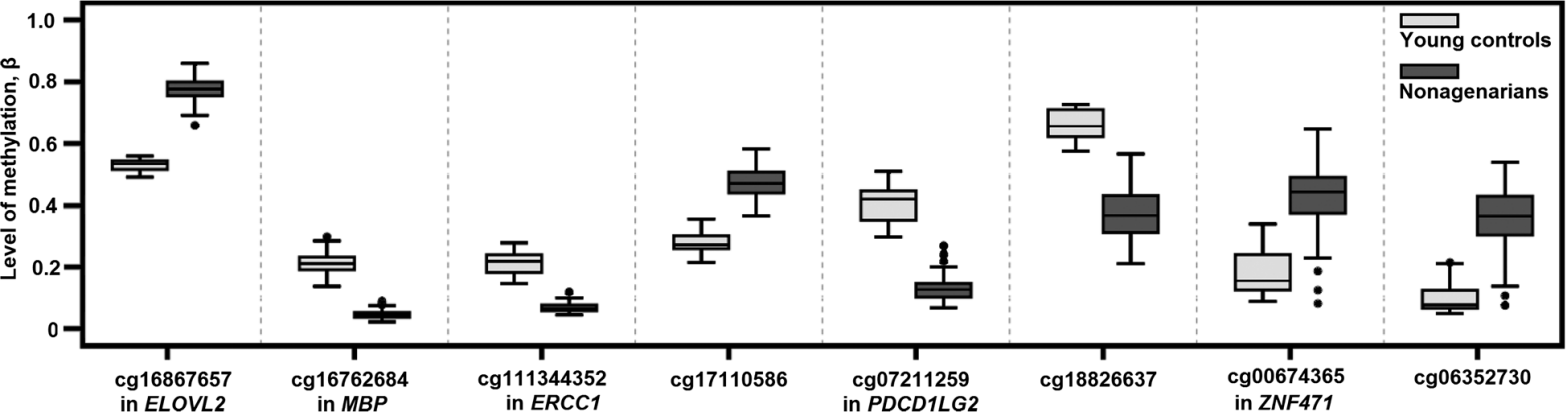
**The top ageing-associated CpG sites.** The level of DNA methylation presented as a box plot in the control and nonagenarian groups and in CpG sites with the strongest association to age group (cg16867657 (*ELOVL2*), cg16762684 (*MBP*), cg111344352 (*ERCC1*) and cg17110586) and in CpG sites with the largest methylation differences (cg07211259 (*PDCD1LG2*), cg18826637, cg00674365 (*ZNF471*) and cg06352730). Gene annotation is shown where applicable. See also Tables 1 and 2.

Among the 8540 ageing-associated CpG sites, 3925 (46%) were hypermethylated, while 4615 (54%) were hypomethylated, in the nonagenarians. The most significant hits, based on the p-values obtained using the site-specific regression models, were cg16867657 (*ELOVL2*), cg16762684 (*MBP*), cg11344352 (*ERCC1*), cg17110586 and cg04875128 (*OTUD7A*). The largest differences in the level of methylation were observed for cg07211259 (*PDCD1LG2*), cg18826637 and cg26063719 (*VIM*), which were hypomethylated in the nonagenarians, and for cg06352730, cg00674365 (*ZNF471*) and cg21402921 (*GABRA5*), which were hypermethylated in the nonagenarians. The top-ranking hits are presented in Tables 1, 2 and Figure 2.

**Table 1:** **Top 10 age-group associated CpG sites from the regression model**

**ProbeID**	**Gene**	**betareg estimate**	**betareg p-value**	**Δβ**	**Wilcoxon p-value**
cg16867657	*ELOVL2*	1.023	6.38E-66	0.243	1.53E-10
cg16762684	*MBP*	-1.486	4.74E-64	-0.168	1.53E-10
cg11344352	*ERCC1*	-1.202	9.15E-63	-0.153	1.53E-10
cg17110586	na	0.895	1.46E-59	0.200	1.53E-10
cg04875128	*OTUD7A*	1.514	7.2E-58	0.279	1.53E-10
cg08262002	*LDB2*	-0.710	2.72E-55	-0.197	1.53E-10
cg18618815	*COL1A1*	-0.941	1.78E-52	-0.225	1.53E-10
cg00748589	na	0.864	1.36E-51	0.179	1.53E-10
cg15416179	*MAP2K3*	-1.131	2.38E-51	-0.187	1.53E-10
cg12065799	*RRAGC*	-0.823	8.15E-51	-0.088	1.53E-10
cg23479922	*MARCH11*	0.940	4.07E-49	0.263	1.53E-10
cg07544187	*CILP2*	1.541	2.35E-48	0.252	1.53E-10
cg09038267	*C10orf26*	1.227	1.48E-47	0.150	1.53E-10
cg13033938	*IP6K1*	-0.699	7.54E-47	-0.061	1.53E-10
cg19283806	*CCDC102B*	-1.253	9.82E-47	-0.267	1.53E-10
cg07547549	*SLC12A5*	0.900	5.02E-46	0.245	1.53E-10
cg01949403	*APOL3*	0.807	7.53E-46	0.111	1.53E-10
cg01243823	*NOD2*	-1.280	7.9E-46	-0.232	1.53E-10
cg22242842	na	-0.952	1.99E-44	-0.206	1.53E-10
cg06007201	*FAM38A*	-0.932	5.65E-44	-0.156	1.53E-10

CpG sites with most significant association to age group in the beta regression models (betareg). To clarify, Δβ refers to difference in the median of DNA methylation values between nonagenarians and young controls (difference in β-value), whereas betareg estimate refers to the estimate obtained from a regression model termed beta regression. Thus the absolute value of betareg estimate and the absolute value of Δβ for a given CpG site are not directly comparable, only the signs of the values are.

**Table 2:** **Top 10 CpG sites with the largest Δβ between nonagenarians and young controls**

**ProbeID**	**Gene**	**betareg estimate**	**betareg p-value**	**Δβ**	**Wilcoxon p-value**
cg07211259	*PDCD1LG2*	-1.086	3.24E-30	-0.290	1.53E-10
cg18826637	na	-1.280	2.23E-32	-0.289	1.53E-10
cg26063719	*VIM*	-1.036	6.09E-25	-0.284	1.53E-10
cg08548498	*SLPI*	-0.767	1.24E-15	-0.278	1.66E-10
cg19283806	*CCDC102B*	-1.253	9.82E-47	-0.267	1.53E-10
cg13591783	*ANXA1*	-0.826	5.17E-22	-0.266	1.53E-10
cg27192248	na	-1.246	2.57E-20	-0.265	1.59E-10
cg03274391	na	-1.263	1.18E-15	-0.264	1.54E-10
cg23654401	*VOPP1*	-0.781	2.94E-16	-0.262	1.54E-10
cg26269881	*BHLHE40*	-1.005	4.25E-25	-0.261	1.53E-10
cg18952796	*NPTX2*	1.121	6.89E-26	0.264	1.56E-10
cg17688525	*L3MBTL4*	0.865	1.36E-11	0.265	5.86E-10
cg27526665	*THRB*	0.940	2.64E-22	0.266	2.0E-10
cg09555124	*IGF2R*	0.944	4.34E-23	0.277	1.53E-10
cg23160016	*GABRA2*	1.041	1.01E-17	0.277	2.49E-10
cg10568066	*RNF39*	0.973	4.68E-13	0.278	1.60E-8
cg04875128	*OTUD7A*	1.514	7.2E-58	0.279	1.53E-10
cg21402921	*GABRA5*	0.868	4.90E-17	0.285	5.58E-10
cg00674365	*ZNF471*	1.033	6.27E-24	0.288	3.65E-10
cg06352730	na	1.437	1.26E-23	0.288	1.76E-10

CpG sites with the largest difference in the methylation level (Δβ) between nonagenarians and controls. To clarify, Δβ refers to difference in the median of DNA methylation values between nonagenarians and young controls (difference in β-value), whereas betareg estimate refers to the estimate obtained from a regression model termed beta regression. Thus the absolute value of betareg estimate and the absolute value of Δβ for a given CpG site are not directly comparable, only the signs of the values are.

### Genomic location of the ageing-associated methylation sites

The ageing-associated CpGs were not uniformly distributed across chromosomes, CpG islands or genes. Chromosomes 2, 3, 4, 5 and 18 contained more ageing-associated methylation sites than expected, whereas chromosomes 16, 17, 19 and 22 had fewer ageing-associated methylation sites than expected (Hypergeometric test p < 0.05, Additional file 2). On the majority of these chromosomes, the proportion of hypermethylated sites compared with the proportion of hypomethylated sites was roughly equal or was slightly biased towards an excess of hypomethylated sites, as in the overall data. Interestingly, on chromosomes 18 and 19, there were considerably more hypermethylated sites than expected: among the identified ageing-associated methylation sites on these chromosomes, 72% and 75% were hypermethylated, constituting a clear overrepresentation compared with the 46% of hypermethylated sites identified in the total data.

**Figure 3:**
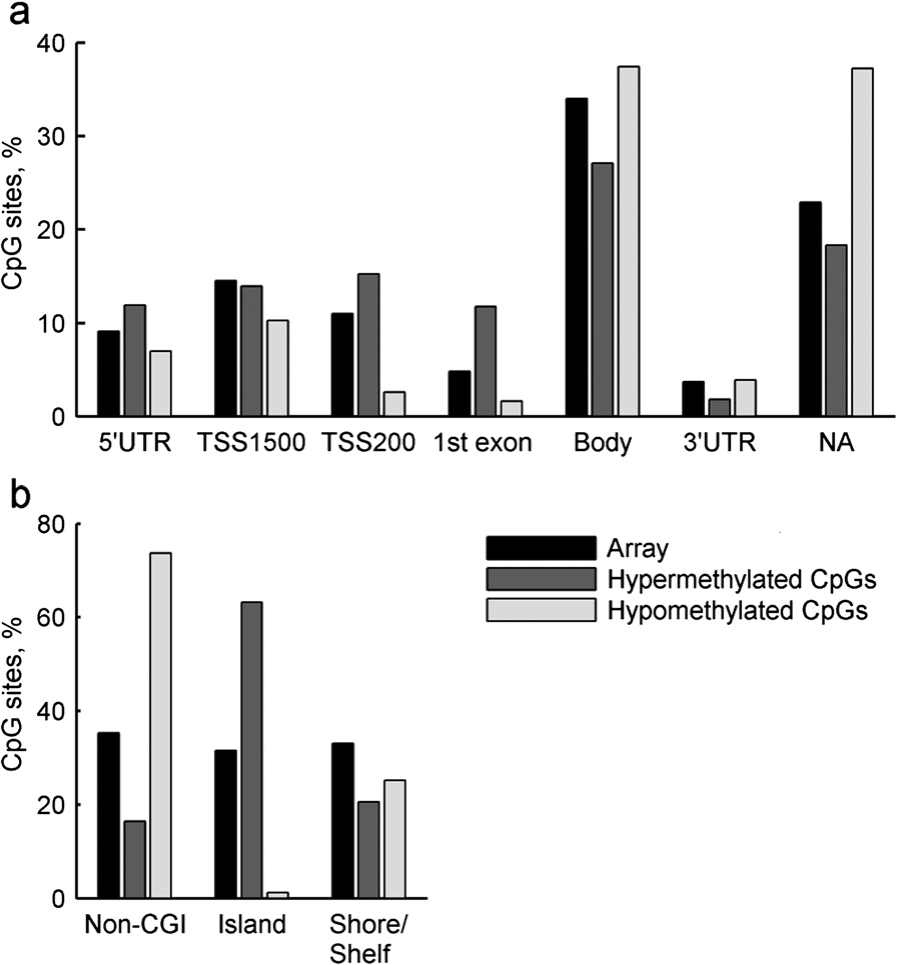
**Locations of the ageing-associated methylation sites identified in the nonagenarians.** Ageing-associated hyper- and hypomethylated probes are distributed differently across the genome. The distribution of ageing-associated CpGs in relation to **(a)** genes and **(b)** CpG islands. Ageing-associated hypermethylation is mainly located in CpG islands, TSSs and the 1^st^ exons of genes, whereas ageing-associated hypomethylation occurs mainly in non-CGIs, gene bodies and areas outside of genes. In the figure, array denotes the distribution of probes in the 450K array.

The CpG sites that were hypermethylated with advancing age were enriched at CpG islands, rather than on island shores or shelves or in non-CGIs. By contrast, the hypomethylated CpG sites were enriched in non-CGIs; their absence from CpG islands was striking, as only 1.2% of all hypomethylated sites were located in CpG islands, whereas 31.5% of the total probes were located in CpG islands (Figure 3). In regard to gene regions, hypermethylated CpGs were enriched in regions near the TSSs and the 1^st^ exons of genes, whereas hypomethylated sites were scarce in these areas and were enriched in the gene body and, more strongly, in the regions outside of genes (Figure 3).

### Functional annotation of the ageing-associated methylation sites

The locations of ageing-associated hyper- and hypomethylation differ, thus it can be assumed that their origins and/or functions also differ. Therefore, we performed the functional analyses separately for hypermethylated and hypomethylated sites and genes harbouring these ageing-associated methylation sites. The 3925 hypermethylated sites were annotated to 1832 different genes, and the 4615 hypomethylated CpG sites were annotated to 2057 different genes.

GOrilla (Gene Ontology enRIchment anaLysis and visuaLizAtion tool) [31,32] was used to identify the GO functions and processes associated with ageing-associated hyper- and hypomethylation-associated genes. For both categories, we identified more significant GO terms for hypermethylation-associated genes, even though there were fewer hypermethylation-associated genes compared with hypomethylation-associated genes. For the hypermethylation-associated genes, 36 enriched GO function terms were identified (Bonferroni corrected p < 0.05), whereas for the hypomethylation-associated genes, 27 enriched GO function terms were identified; 11 of these terms were common to the two groups (Additional file 3). The top GO function terms for the hypermethylation-associated genes were unique to these genes; these terms were associated with sequence-specific DNA binding and transcription factor binding (also presented as a diagram in Additional file 4). The GO terms that were enriched only for hypomethylated sites did not reveal similar enrichment for a common process (Additional file 4). The GO function terms that were common to hypermethylation- and hypomethylation-associated genes also formed a group and were clustered around channel function-associated GO terms. The results for GO process terms was similar to that for GO functions, as we identified 265 significant GO terms for hypermethylation-associated genes, whereas for hypomethylation-associated genes, we identified only 53 significant GO terms; 41 of these terms were common to hyper- and hypomethylation-associated genes. (Additional file 5). The top-ranking hypermethylation-specific GO terms were clustered around two types of processes: development and morphogenesis; and metabolic processes, gene expression and nucleotide metabolism (Additional file 6). Again, the significant GO terms associated with hypomethylation did not belong to a specific group (Additional file 6). The hypermethylation-specific GO terms that formed specific clusters are presented in Tables 3, 4 and 5.

**Table 3:** **Hypermethylation-specific GO function terms in nonagenarians**

**GO term**	**Description**	**P-value**	**FDR q-value**	**Rank (out of 36)**
GO:0043565	Sequence-specific DNA binding	1.18E-32	4.65E-29	1
GO:0001071	Nucleic acid binding transcription factor activity	9.38E-31	1.85E-27	2
GO:0003700	Sequence-specific DNA binding transcription factor activity	2.22E-30	2.92E-27	3
GO:0003677	DNA binding	6.48E-16	6.38E-13	4
GO:0000981	Sequence-specific DNA binding RNA polymerase II transcription factor activity	2.6E-15	2.05E-12	5
GO:0000976	Transcription regulatory region sequence-specific DNA binding	4.8E-13	2.7E-10	7
GO:0044212	Transcription regulatory region DNA binding	7.92E-12	3.47E-9	9
GO:0000975	Regulatory region DNA binding	2.22E-11	8.75E-9	10
GO:0001067	Regulatory region nucleic acid binding	2.22E-11	7.96E-9	11

This table includes only the hypermethylation-specific GO function terms that form a common cluster, associated with DNA binding and transcription. The presented p-values are unadjusted and the threshold for significance is 1.27e-5 (Bonferroni). The rank denotes the placement of a given GO term in the list of all significant GO terms. For all statistically significant GO function terms, see Additional file 3.

**Table 4:** **Hypermethylation-specific GO process terms in nonagenarians**

**GO term**	**Description**	**P-value**	**FDR q-value**	**Rank (out of 265)**
GO:0048598	Embryonic morphogenesis	1.25E-22	8.85E-20	17
GO:0048729	Tissue morphogenesis	2.99E-19	1.64E-16	22
GO:0002009	Morphogenesis of an epithelium	6.94E-18	3.22E-15	26
GO:0001763	Morphogenesis of a branching structure	1.84E-17	7.65E-15	29
GO:0048754	Branching morphogenesis of an epithelial tube	1.26E-15	3.09E-13	49
GO:0048562	Embryonic organ morphogenesis	6.12E-14	1.1E-11	67
GO:0009887	Organ morphogenesis	1.13E-13	1.92E-11	71
GO:0035107	Appendage morphogenesis	2.17E-12	2.85E-10	92
GO:0035108	Limb morphogenesis	2.17E-12	2.82E-10	93
GO:0030326	Embryonic limb morphogenesis	7.07E-12	8.7E-10	98
GO:0035113	Embryonic appendage morphogenesis	7.07E-12	8.61E-10	99
GO:0048704	Embryonic skeletal system morphogenesis	2.25E-11	2.56E-9	106
GO:0048705	Skeletal system morphogenesis	1.04E-10	1.11E-8	113
GO:0048732	Gland development	2.37E-10	2.3E-8	124

This table includes only the hypermethylation-specific GO process terms that form a common cluster, associated with development and morphogenesis. The presented p-values are unadjusted and the threshold for significance is 4.15e-6 (Bonferroni). The rank denotes the placement of a given GO term in the list of all significant GO terms. For all statistically significant GO process terms, see Additional file 5.

**Table 5:** **Hypermethylation-specific GO process terms in nonagenarians**

**GO term**	**Description**	**P-value**	**FDR q-value**	**Rank (out of 265)**
GO:0045935	Positive regulation of nucleobase-containing compound metabolic process	7.07E-17	2.37E-14	36
GO:0051173	Positive regulation of nitrogen compound metabolic process	1.06E-16	3.44E-14	37
GO:0031328	Positive regulation of cellular biosynthetic process	2.74E-16	8.47E-14	39
GO:0009891	Positive regulation of biosynthetic process	3.61E-16	1.06E-13	41
GO:0045893	Positive regulation of transcription, DNA-templated	5.05E-16	1.35E-13	45
GO:0019219	Regulation of nucleobase-containing compound metabolic process	7.72E-16	2.02E-13	46
GO:0010628	Positive regulation of gene expression	1.11E-15	2.78E-13	48
GO:0006357	Regulation of transcription from RNA polymerase II promoter	5.05E-15	1.15E-12	53
GO:0031326	Regulation of cellular biosynthetic process	1.07E-14	2.27E-12	57
GO:0051171	Regulation of nitrogen compound metabolic process	1.28E-14	2.66E-12	58
GO:0009889	Regulation of biosynthetic process	1.45E-14	2.96E-12	59
GO:0051254	Positive regulation of RNA metabolic process	1.8E-14	3.56E-12	61
GO:0006355	Regulation of transcription, DNA-templated	1.98E-14	3.85E-12	62
GO:1902680	Positive regulation of RNA biosynthetic process	2.06E-14	3.93E-12	63
GO:0045944	Positive regulation of transcription from RNA polymerase II promoter	2.94E-14	5.45E-12	65
GO:0010557	Positive regulation of macromolecule biosynthetic process	6.72E-14	1.19E-11	68
GO:0031323	Regulation of cellular metabolic process	1.09E-13	1.87E-11	70
GO:2001141	Regulation of RNA biosynthetic process	1.37E-13	2.29E-11	72
GO:0031325	Positive regulation of cellular metabolic process	2.22E-13	3.57E-11	75
GO:0045934	Negative regulation of nucleobase-containing compound metabolic process	2.8E-13	4.39E-11	77
GO:0031327	Negative regulation of cellular biosynthetic process	3.75E-13	5.8E-11	78
GO:0009893	Positive regulation of metabolic process	3.84E-13	5.85E-11	79
GO:0009890	Negative regulation of biosynthetic process	3.84E-13	5.78E-11	80
GO:0000122	Negative regulation of transcription from RNA polymerase II promoter	5.25E-13	7.72E-11	82
GO:0051252	Regulation of RNA metabolic process	9.69E-13	1.39E-10	84
GO:0051172	Negative regulation of nitrogen compound metabolic process	1.07E-12	1.52E-10	85
GO:2000112	Regulation of cellular macromolecule biosynthetic process	1.14E-12	1.6E-10	86
GO:0080090	Regulation of primary metabolic process	1.39E-12	1.92E-10	87
GO:0010629	Negative regulation of gene expression	2.02E-12	2.68E-10	91
GO:0010556	Regulation of macromolecule biosynthetic process	3.09E-12	3.96E-10	94
GO:0045892	Negative regulation of transcription, DNA-templated	4.17E-12	5.29E-10	95
GO:1902679	Negative regulation of RNA biosynthetic process	4.83E-12	6.07E-10	96
GO:0019222	Regulation of metabolic process	1.56E-11	1.84E-9	102
GO:0051253	Negative regulation of RNA metabolic process	1.94E-11	2.25E-9	104
GO:0010468	Regulation of gene expression	1.35E-10	1.39E-8	117
GO:0010558	Negative regulation of macromolecule biosynthetic process	1.39E-10	1.41E-8	119
GO:0010604	Positive regulation of macromolecule metabolic process	1.49E-10	1.46E-8	123
GO:2000113	Negative regulation of cellular macromolecule biosynthetic process	3.62E-10	3.38E-8	129

This table includes only the hypermethylation-specific GO process terms that form a common cluster, associated with nucleotide metabolism, RNA metabolism and transcription. The presented p-values are unadjusted and the threshold for significance is 4.15e-6 (Bonferroni). The rank denotes the placement of a given GO term in the list of all significant GO terms. For all statistically significant GO process terms, see Additional file 5.

PScan [33] was used to identify transcription factors that could be common regulators of the identified genes. For the hypermethylation-associated genes, 24 common transcription factors were identified (Additional file 7), whereas for the hypomethylation-associated genes, only one TF (EWSR1-FLI1, p-value 1.502e-5), was identified. Among the 24 transcription factors that were common to hypermethylation-associated genes, half (12) were zinc-coordinating transcription factors.

**Figure 4:**
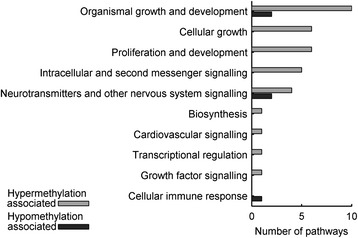
**Canonical pathway categories associated with differentially methylated genes in the nonagenarians.** Hypomethylation-associated genes are enriched in only three canonical pathways, thus corresponding to only a few pathway categories. Hypermethylation-associated genes are enriched in canonical pathways associated mainly with organismal and cellular growth and development. One canonical pathway can belong to several categories; for the individual pathways, see Additional file 8.

We also identified canonical pathways related to hypo- and hypermethylation-associated genes through Ingenuity pathway analysis (IPA) [34]. For the hypermethylation-associated genes, we identified 19 affected canonical pathways (Benjamini-Hochberg-corrected p-value <0.05), whereas for the hypomethylation-associated genes, 3 pathways were identified, 1 of which was common to both groups of genes (Additional file 8). The canonical pathways associated with hypermethylation in nonagenarians belonged to signalling pathway categories such as *Organismal growth & development*, *Cellular growth* and *Proliferation and development* (Figure 4).

### Effect of sex on ageing-associated DNA methylation changes

Among the 8540 ageing-associated, high-confidence CpG sites, only 7 showed a statistically significant association with sex in our beta regression analysis in which age group, sex and leukocyte proportions were included as covariates. The sex-associated sites that were also ageing-associated are listed in Additional file 9.

### Association between ageing-associated DNA methylation changes and gene expression

We performed a correlation analysis between the level of methylation at ageing-associated CpGs and the expression level of genes in which these CpG sites were located. In nonagenarians, we identified 422 correlation pairs (Pearson correlation, Benjamini-Hochberg-corrected p-value < 0.05) that consisted of 377 individual CpG sites and 233 individual genes (Additional file 10). The apparent discrepancy in these numbers is because a single CpG can be located in a region of overlapping transcripts, and there can be several CpGs within the coding region of a single transcript. In the young controls, we identified 50 expression-methylation correlation pairs (Pearson correlation, Benjamini-Hochberg-corrected p-value < 0.05), consisting of 43 individual CpGs and 37 individual genes. In nonagenarians, 255 (60%) of these correlated CpG-gene pairs showed an inverse correlation, and 167 (40%) exhibited a direct correlation. In the young controls, these numbers were 46 (92%) and 4 (8%), respectively. Among the genes whose expression level was correlated with the level of DNA methylation, 23 were common to the nonagenarians and young controls, and in all cases, the direction of correlation was the same. We previously showed that 14 of the 233 genes identified in the present study were differentially expressed with age in both sexes and that an additional 14 were differentially expressed with age in either sex [35] (For details, see Additional file 10).

The correlated CpGs did not exhibit a similar distribution in the genome to the ageing-associated methylation sites. Those CpG sites whose methylation level correlated with the level of gene expression were concentrated in non-CGIs and on CpG island shores and shelves, whereas only a few (14.6%) were located in CpG islands. In the non-CGIs, the majority of correlations were direct, whereas the opposite situation was observed in CpG islands and on island shores and shelves. With regard to regions within genes, the correlated CpGs were relatively evenly distributed. However, we identified an abundance of correlated CpGs within gene bodies (55% of all correlated sites), where the majority of sites were directly correlated. In regions near a TSS (TSS200, from TSS to -200 nucleotides upstream of TSS), directly correlated CpGs were almost completely absent (Additional file 11).

To analyse the processes associated with the genes that displayed a correlation between expression and methylation levels, we performed GO term analysis and IPA for the nonagenarians. We identified 20 GO process terms (Bonferroni-corrected p-value <0.05), of which 6 (30%) were immune system associated. Numerous immune system pathways were also identified when considering GO process terms that were more loosely associated with these genes (Benjamini-Hochberg-corrected p-value <0.05), where 39 of 121 (32%) statistically significant GO process terms were immune system associated (Additional file 12). Only one GO function term (GO:0005515 Protein binding) was associated with the correlated CpGs. In addition to the immune system, pathways related to the reaction to the environment were affected. Ingenuity canonical pathway analysis revealed 15 canonical pathways (Benjamini-Hochberg-corrected p-value <0.05) (Table 6), the majority of which were directly immune system associated (*Crosstalk between Dendritic Cells and Natural Killer Cells*, *Antigen Presentation Pathway*, *Fcγ Receptor-mediated Phagocytosis in Macrophages and Monocytes*, *T Helper Cell Differentiation*) or associated with cytoskeleton remodelling and endocytosis (*Integrin Signalling*, *Actin Cytoskeleton Signalling*, *Tec Kinase Signalling*, *Paxillin Signalling*, *Caveolar-mediated Endocytosis Signalling*).

**Table 6:** **Canonical pathways associated with genes whose expression levels correlate with the level of DNA methylation in nonagenarians**

**Ingenuity canonical pathways**	**p-value (B-H corrected)**	**Ratio**	**Molecules**
Integrin signalling	0.016	0.054	ITGB1,PTK2,RAP2A,FYN,PAK1,RALA,ACTA2,ITGA6,CAPN2,ITGAL,ACTN1
Actin cytoskeleton signalling	0.017	0.048	ITGB1,PTK2,TIAM1,PAK1,F2R,ACTA2,TRIO,PDGFD,GSN,ARHGAP24,ACTN1
Tec kinase signalling	0.019	0.051	STAT4,ITGB1,PTK2,FYN,GNAI3,GNB4,PAK1,ACTA2,HCK
Agrin interactions at neuromuscular junction	0.019	0.090	ITGB1,PTK2,PAK1,ACTA2,ITGA6,ITGAL
Paxillin signalling	0.020	0.064	ITGB1,PTK2,PAK1,ACTA2,ITGA6,ITGAL,ACTN1
Reelin signalling in neurons	0.026	0.073	ITGB1,FYN,HCK,ITGA6,ARHGEF11,ITGAL
Phospholipase C signalling	0.030	0.041	ITGB1,FYN,GNB4,RALA,AHNAK,SYK,MEF2C,ARHGEF11,PLD6,CREB5
Germ cell-sertoli cell junction signalling	0.030	0.051	ITGB1,PTK2,TGFBR2,PAK1,ACTA2,ITGA6,GSN,ACTN1
Crosstalk between dendritic cells and natural killer cells	0.030	0.066	IFNG,ACTA2,CD86,HLA-F,ITGAL,CCR7
Protein kinase A signalling	0.030	0.035	TGFBR2,PTK2,GNB4,GNAI3,TCF4,PTPN7,YWHAG,DUSP10,RYR1,LEF1,CREB5,PTPRM,SIRPA
Antigen presentation pathway	0.030	0.100	PSMB9,IFNG,HLA-F,HLA-DPB1
Fcγ receptor-mediated phagocytosis in macrophages and monocytes	0.030	0.063	FYN,PAK1,SYK,ACTA2,HCK,PLD6
T helper cell differentiation	0.037	0.073	STAT4,TGFBR2,IFNG,IFNGR2,CD86
Ephrin receptor signalling	0.038	0.041	ITGB1,PTK2,FYN,GNAI3,GNB4,PAK1,PDGFD,CREB5
Caveolar-mediated endocytosis signalling	0.044	0.062	ITGB1,FYN,ACTA2,ITGA6,ITGAL

Canonical pathways (IPA [34]) associated with genes whose expression levels correlate with the level of DNA methylation. P-values are Benjamini-Hochberg corrected. Ratio = number of identified genes/number of genes in the pathway. Molecules refer to genes affected in our analysis present in the given pathway.

## Discussion

### Ageing-associated DNA methylation changes; single CpG sites and their location and function

Here, we present the results of our ageing-associated DNA methylation analysis. In summary, our results were obtained with a 450K array using PBMCs collected from nonagenarians and young controls. The study subjects were analysed as two age groups, and we used two different statistical methods to verify that the ageing-associated methylation sites identified had a prominent difference in the level of methylation between the age groups and that this difference was not due to changes in leukocyte proportions. The proportions of leukocytes were measured via FACS. We also added a layer of information by including gene expression data from the same individuals. The small number of young controls is a potential limitation in our study; thus, the results should be interpreted accordingly.

In previous ageing-methylation studies, the age range of the oldest study subjects has typically been from 70 to 80 years of age [17,19-22,25,26], and the youngest age group to be included has ranged from new-borns [22,28] to 50 years of age [20,25]. In studies in which subjects over 90 years old have been analysed, these individuals represented a minority of the study population or the overall sample size has been small [18,27,28,36]. Hence, a strength of our study is the large number of the oldest-old individuals homogenous in terms of age. In addition, our study population represents the two extremities of adulthood, and as age was used as a dichotomous variable we were able to identify both changes occurring linearly with age as well as changes that occur in either end of the spectrum. The DNA methylation studies performed with 27K arrays [17-22] fail to capture methylation changes outside gene promoters, yet our results, as well as those of others [25,28,36], show that ageing-associated changes are not restricted to gene promoters. In contrast to our study, previous reports combining methylation and expression data have relied on individuals from different study cohorts [27,36]. A group of studies have also tried to identify a small set of methylation sites that could be used to construct an ageing signature [22,23,25,27]. However, by focusing on a broader set of ageing-associated methylation sites, the mechanisms of ageing can be more thoroughly examined. Given that published ageing-methylation studies have been conducted using various age ranges and statistical methods, discrepancies in the results are most likely due to both biological and statistical factors.

The main characteristics of the ageing-associated methylation sites identified in the present study are presented in Table 7. We identified 8540 high-confidence CpG sites that show a large difference in methylation levels between nonagenarians and young controls and that present high statistical significance in a regression model adjusted for the leukocyte proportion. A slight majority (54%) of the identified sites were hypomethylated in the nonagenarians. Among the top-ranking ageing-associated methylation changes that have been reported with a high frequency, *ELOVL2* (cg16867657, cg24724428), *PENK* (cg04598121), *FHL2* (cg22454769, cg24079702, cg06639320) *EDARADD* (cg09809672), *KLF14* (cg04528819, cg07955995) and *OTUD7A* (cg04875128) were also identified in our study. Of these genes, only *EDARADD* was hypomethylated in the nonagenarians compared with the controls. As reported by Steegenga et al. [24], among 8 previous studies analysing the association of ageing and DNA methylation changes in PBMCs [17,19-21,25-28], only 529 probes were reported to be affected by age by more than one research group. Of these probes, our analysis identified 105. Interestingly, the majority of frequently reported CpG sites are hypermethylated with increasing age. Among the 151 CpG sites (148 of which are present in 450K) reported to be associated with ageing by more than 3 groups [24], 77% were hypermethylated. Of the 105 CpG sites that are frequently reported and were identified in our study, 79% (83/105) were hypermethylated.

**Table 7:** **Characteristics of ageing-associated methylation sites**

	**Hypermethylated**	**Hypomethylated**
n	3925	4615
CpG island location	CpG islands	Non-CGI
Genomic location	TSS, 1st exon	Gene body, outside genes
Associated genes	1832	2057
GO function terms	36	27
GO process terms	265	53
Canonical pathways (IPA)	19	3
Transcription factors	24	1

The CpG island location and genomic location refer to the sites where hyper- and hypomethylated sites are most abundant. Notably, there are more hypomethylated CpG sites compared with hypermethylated CpG sites and therefore also more hypomethylation-associated genes, yet the hypermethylation-associated genes are enriched in more GO terms and canonical pathways, and they share more common transcription factors.

The functional roles of the 10 most frequently reported ageing-associated methylation sites are currently unclear, as they are not associated with a common, ageing-related process. According to our results, the genes associated with these sites are not enriched under a common GO term or in common canonical pathways. Only *FHL2*, *PENK* and *OTUD7A* are included in any identified GO term, and none of them are included in affected canonical pathways. The methylation levels of frequently reported CpGs are not correlated with the expression levels of the corresponding genes. For *EDARADD*, we identified an additional CpG site (cg18964582) that was differentially methylated between nonagenarians and young controls, located within TSS1500, where there is an inverse correlation between the methylation level and the expression level. However, based on previous findings and our results, it appears that the frequently reported ageing-associated CpG sites are not strongly associated with known ageing-related mechanisms but could instead represent a cellular chronological clock mechanism.

Our results revealed an enrichment of ageing-associated hypermethylation at CpG islands, whereas hypomethylation was enriched in non-CGIs and was almost totally absent from CpG islands. These findings are in line with previously reported results [16,24,25,28,36,37]. The majority of CpG sites are not initially methylated in CpG islands, and the change observed during ageing is hypermethylation. The opposite is true for regions with few CpG sites that initially are heavily methylated, and the non-CGIs are associated with hypomethylation. These results support the notion that the normal maintenance of DNA methylation patterns is disrupted with ageing [38]. As both hypomethylation and hypermethylation occur with ageing, it appears that both *de novo* methylation processes, mediated by *DNMT3A* and *DNMT3B* methyltransferases, and the maintenance of existing DNA methylation, mediated by *DNMT1*, are disrupted with ageing. Interestingly, our results identified 4 CpG sites in *DNMT3A* that were ageing associated (cg00050692, which was hypomethylated, and cg15302376, cg15843262 and cg26544247, which were hypermethylated in the nonagenarians). However, there was no correlation between the level of methylation and *DNMT3A* expression.

Our results showed that not only are ageing-associated hyper- and hypomethylation found at different genomic sites but that these changes are also found in genes associated with different functions. Our findings further revealed that ageing-associated hypermethylation is concentrated in genes associated with developmental processes as well as DNA-binding and transcription of genes, whereas hypomethylation is not enriched among a specific set of genes. Johansson et al. [36], Rakyan et al. [19] and Florath et al. [25] previously reported the association of hypermethylation with developmental processes and DNA binding. As DNA methylation regulates DNA transcription, it is interesting that the genes required during this process are differentially methylated with ageing. In comparison with ageing-associated hypomethylation, hypermethylation appears to be a more regulated process, as no strongly hypomethylation-specific functions or processes were identified in this study.

It is notable that while the individual sites reported to be ageing associated differ to some extent between studies, the results regarding their locations in the genome and the molecular functions with which they are associated are more uniform. Single highly significant CpG sites have also been reported in various studies, including sites located in the *ELOVL2* and *FLH2* genes. Common ageing-associated DNA methylation changes can also be observed across different tissues [6,23]. Thus, it appears that at least some fraction of ageing-associated DNA methylation changes is caused by programmed or pseudo-programmed changes that occur in a similar manner across tissues and individuals. As certain processes and sites are reported frequently, it can be hypothesised that these sites and processes represent clock-like changes associated with ageing. For example, a strong association with chronological age has been shown for *ELOVL2* (cg16867657) [25,26,36]. However, it remains to be investigated whether these sites are only associated with chronological age or if there are also associations with phenotypic changes related to (successful) ageing. If these frequently reported sites are only markers of chronological age, markers of biological age are yet to be identified.

### The role of cell proportions in DNA methylation studies

The majority of DNA methylation and expression studies are performed with whole blood or PBMCs due to the accessibility of these tissue types. However, PBMCs consist of various cell types, and different individuals can exhibit differences in the proportions of different cell populations. Ageing is known to be associated with changes in the proportions of T cells [2,39]. Furthermore, the different leukocyte subtypes show differences in their DNA methylation levels [40], and changes in DNA methylation are known to be one of the factors regulating lineage development in leukocytes [41].

Previous reports have claimed that differences in the proportions of leukocytes do not cause bias in methylation analyses [17,21,22]. However, contradictory reports have also been published [42], and recently it has been systematically shown that differences in leukocyte proportions should be taken into consideration when analysing ageing-associated methylation differences [29]. Our PCA revealed that the largest percentage of the variation in our methylation data was associated with the proportions of different leukocyte subtypes (Figure 1).

### The role of sex in ageing-associated DNA methylation studies

According to our results, sex does not have a large effect on ageing-associated DNA methylation changes in autosomes, as we identified only 7 CpG sites for which sex, in addition to age group, was a significant covariate in the regression model. However, the small number of male samples in our control population may have precluded the identification of ageing-associated sex differences. Nevertheless, Johansson et al. [36] and McClay et al. [37] previously reported similar findings in studies focusing on individual sites associated with ageing. In studies where methylation profiles have been used to predict age, however, the methylome has been shown to age more rapidly in men than in women [22,27]. DNA methylation is believed to mediate the long-term regulation of gene expression [13], and it is therefore interesting to note that sex differences appear to be mediated via mechanisms other than DNA methylation. Apparently, the effects of sex observed in methylome studies predicting age are small global effects rather than large changes at a limited number of sites. We have previously reported [35] that there are sex-specific differences in the gene expression changes associated with ageing, but based on the results of the present study, these expression differences are not regulated by DNA methylation.

### The role of zinc-associated proteins in ageing

We observed a clear enrichment of hypermethylation on chromosome 19, which seems to be due to the abundance of zinc finger proteins on this chromosome. The increased methylation of zinc finger genes on chromosome 19 has previously been observed in oropharyngeal squamous cell carcinoma [43], and similarities between the methylation changes that occur in ageing and cancer have been demonstrated in multiple studies [20,21,23]. It has recently been proposed that the zinc finger proteins on chromosome 19 have specifically evolved to repress endogenous retroviruses (ERVs) [44]. On the other hand, the expression of ERVs has been associated with ageing in mice [45,46]. Hence, the hypermethylation of zinc finger genes observed with ageing offers an explanation for why ERVs are able to be expressed with advancing age. One of the zinc finger genes predicted to repress ERVs by Lukic et al. [44] was *ZNF154*. We identified 10 CpGs within this gene as being hypermethylated in the nonagenarians, and we identified a strong negative correlation between the level of methylation and the expression of this gene, indicating that its expression is truly downregulated in the aged individuals. Both ageing and cancer are associated with genomic instability [1], and the role of active ERVs in inducing this genomic instability with increasing age could be analogous to that proposed in cancer [47].

Zinc-coordinating transcription factors were also enriched among the TFs predicted to regulate hypermethylation-associated genes in this study, as 12 out of the 24 identified TFs were zinc coordinating. Zinc has been associated with various processes that are known to be regulated during ageing, such as immune function, DNA repair mechanisms, cell proliferation, apoptosis and transcription [48,49].

### The association between ageing-associated DNA methylation changes and gene expression

We sought to examine the relationship between ageing-associated DNA methylation changes and gene expression levels. Compared with previous studies, a key asset of our study is that methylation and gene expression data were available from the same samples. Those ageing-associated methylation sites in which the level of methylation is associated with the level of gene expression are concentrated in non-CGIs and on shores and shelves, as well as in gene body regions. Similar findings have been reported by Zilbauer et al. [40]. Gene-body methylation has been proposed to affect gene expression via splicing and alternative start site usage [13,50]. It is important to note that many previous studies examining DNA methylation changes during ageing have been performed using the Illumina 27K array, where the majority of the probes are located in promoter regions. In these studies, the effects of gene-body methylation on gene expression levels remained unidentified.

The identified genes that display expression-methylation correlations are strongly enriched in immunological processes and in cytoskeletal remodelling and endocytosis. Cytoskeletal remodelling is required for leukocyte activation, migration and phagocytosis [51]. The results imply that some fraction of ageing-associated immune system changes may be regulated by DNA methylation. Defects in the immune system are a hallmark of ageing, leading to increased susceptibility to infectious diseases, cancer and ultimately death [1]. DNA methylation typically regulates long-term trends in gene expression [11,13], and the possibility that immune system-related processes may be locked in a particular state by DNA methylation could offer one explanation as to why the immune system of elderly individuals is not able to respond appropriately to various insults.

We found that only a minority of ageing-associated CpG sites showed an association between methylation and expression levels. Furthermore, only a minority of these genes have been identified as differentially expressed between nonagenarians and young individuals [35]. Previous studies have also found a limited number of associations between ageing-associated DNA methylation changes and gene expression levels [21,23,27,36,42,52]. Due to the methods applied in the present study, not all the effects of DNA methylation on gene expression could be detected; this limitation is also true for previously reported results. The textbook case of DNA methylation regulating gene expression (the methylation of a promoter and silencing of a gene) remains undetected in many cases because in an array analysis, an unexpressed gene shows no signal that can be distinguished from background and is therefore typically omitted from the analysis. Additionally, in the present study, the methylation level of each CpG was correlated separately with gene expression. In CpG island regions in particular, the effect of DNA methylation changes on gene expression could be observed when a cluster of closely located CpG sites were analysed as a whole. The effects of CpG sites that are not located in the regulated gene itself also remain unidentified. The short list of methylation-gene expression associations linked to ageing reported herein and previously by others should be interpreted as a defined set of one type of methylation-gene expression associations, and it should be assumed that other types of mechanisms exist and require different methodologies to be identified.

## Conclusions

Based on the results presented here, it appears that ageing-associated hyper- and hypomethylation are distinct processes, both in terms of their causes and consequences. We suggest that hypermethylation is an active process, caused by programmed or pseudo-programmed ageing processes, and that hypermethylation is strongly associated with chronological age. Ageing-associated hypomethylation, however, is a passive process caused by stochastic or environmental effects and is associated with biological age, i.e., the phenotype of the individual. Whether the underlying cause of ageing is programmed, pseudo-programmed or due to the accumulation of molecular damage has been widely discussed in the literature. Given that evidence supporting each theory can be found, it is plausible that these mechanisms all contribute to the ageing process but possibly affect different aspects [3-5].

First, hypermethylation is an active process that consumes energy as new methyl groups are added to DNA by DNA methyltransferases. Hypomethylation can also be an active process in some cases, but contrary to hypermethylation, it may occur passively as well [53,54]. The most frequently reported ageing-associated DNA methylation changes (for example in *ELOVL2*) that are repeated across tissues and study populations, thus implying programmed changes, are hypermethylation events. In studies where chronological age has been explained in association with DNA methylation levels, it has been found that at sites showing the strongest correlation with chronological age, methylation increases with age [25,26]. The ageing-associated hypermethylated sites form common groups with regard to cellular processes and functions. According to the results of the present study, hypermethylation-associated genes are predicted to be regulated by a common group of transcription factors and are also enriched in common GO terms, whereas hypomethylation-associated genes do not to appear to form common groups. The top-ranking ageing-associated sites are hypermethylated, but hypomethylated sites are more numerous. This difference becomes more significant when the threshold of significance is lowered; of the 8540 sites identified here, 54% were hypomethylated, but among the 45507 sites identified with the regression model, 64% were hypomethylated. Johansson et al. [36] also reported an excess of hypomethylation over hypermethylation with ageing.

Global hypomethylation has been associated with an increasing risk of frailty [55], but very few other associations between phenotype and DNA methylation have been reported [17]. However, this may be due to technical concerns, as the study by Bell et al. [17] was performed with the 27K array, which almost exclusively contains promoter-associated probes that are not methylated at baseline and can therefore primarily acquire hypermethylation. Phenotype association studies performed with the 450K array or using sequencing techniques are necessary to clarify if hypomethylation is associated with typical ageing-associated phenotypes.

The role of DNA methylation is known to differ depending on its location in the genome. Thus, it would not be surprising if different DNA methylation changes in the genome are affected by different ageing mechanisms. As DNA methylation analyses are complicated by the different effects of methylation sites at different genomic positions and by the cumulative effects of nearby CpG sites, all possible known biases, such as the proportions of leukocytes, should be accounted for in DNA methylation analyses.

## Methods

### Study population

The study population consisted of 146 nonagenarians (females n = 103, males n = 43) participating in the Vitality 90+ study and 30 young, healthy controls (aged 19-30 years, median 22.5 years; females n = 21, males n = 9). Gene expression data were available for all the individuals, and methylation data were available for 122 nonagenarians (n = 89 females and n = 33 males) and 21 young controls (n = 14 females and n = 7 males), and data on cell proportions were available for 115 nonagenarians (n = 84 females and n = 31 males) and all 30 of the young controls. All the study subjects were of Western European descent. The Vitality 90+ study is an on-going prospective population-based study that includes both home-dwelling and institutionalised individuals aged 90 years or more who live in the city of Tampere, Finland. The recruitment and characterisation of the participants were performed as previously reported for earlier Vitality 90+ study cohorts [56]. In this study, we included only individuals born in 1920, and the evaluated samples were collected in the year 2010. The nonagenarians included in the study had not had any infections or received any vaccinations in the 30 days prior to blood sample collection. The young controls consisted of healthy laboratory personnel who did not have any medically diagnosed chronic illnesses, were non-smokers and had not had any infections or received any vaccinations within the two weeks prior to blood sample collection. The study participants provided their written informed consent. The study has been conducted according to the principles expressed in the declaration of Helsinki, and the study protocol was approved by the ethics committee of the city of Tampere (1592/403/1996).

### Sample collection

The blood samples were collected into EDTA-containing tubes by a trained medical student during a home visit. All the blood samples were drawn between 8 am and 12 am. The samples were directly subjected to leucocyte separation on a Ficoll-Paque density gradient (Ficoll-Paque™ Premium, cat. no. 17-5442-03, GE Healthcare Bio-Sciences AB, Uppsala, Sweden). The PBMC layer was collected, and a subset of the cells was suspended in 150 μl of RNAlater solution (Ambion Inc., Austin, TX, USA) for use in a gene expression microarray analysis. Cells that were to be subjected to FACS analysis and DNA extraction were suspended in 1 ml of a freezing solution (5/8 FBS, 2/8 RPMI-160 medium, 1/8 DMSO) (FBS cat. no. F7524, Sigma-Aldrich, MO, USA; RPMI: cat. no. R0883, Sigma-Aldrich, MO, USA; DMSO: cat. no. 1.02931.0500, VWR, Espoo, Finland).

### DNA extraction

DNA was extracted from PBMCs using the QIAamp DNA Mini kit (Qiagen, CA, USA), following the manufacturer’s instructions for the spin protocol. The DNA was eluted in 60 μl of AE elution buffer and stored at -20°C. The concentration and quality of the DNA was assessed with the Qubit dsDNA HS Assay (Invitrogen, Eugene, OR, USA).

### RNA extraction

For RNA extraction, equal amounts of PBS and RNAlater were added to the cell suspension and then removed via centrifugation, leaving only the cell pellet. RNA was purified using an miRNeasy mini kit (Qiagen, CA, USA), according to the manufacturer’s protocol, with on-column DNase digestion (AppliChem GmbH, Darmstadt, Germany). The concentration and quality of the RNA were assessed with the Agilent RNA 6000 Nano Kit on an Agilent 2100 Bioanalyzer (Agilent Technologies, CA, USA).

### FACS

The proportions of different lymphocyte populations were determined through FACS analysis (BD FACSCanto II), and the results were analysed with BD FACS Diva, version 6.1.3 (BD Biosciences, Franklin Lakes, NJ, USA). The antibodies employed in this analysis were FITC-CD14 (cat. no. 11-0149), PerCP-Cy5.5-CD3 (45-0037), APC-CD28 (17-0289) (eBioscience, San Diego, CA, USA), PE-Cy™7-CD4 (cat. no. 557852) and APC-Cy™7-CD8 (557834) (BD Biosciences).

### Expression array

Labelled cRNA was prepared from 330 ng of total RNA using the Illumina TotalPrep RNA Amplification Kit (Ambion Inc., TX, USA) with overnight incubation according to the manufacturer’s protocol. The quality of the labelled cRNA was determined using a 2100 Bioanalyzer (Agilent Technologies). In total, 1500 ng of labelled cRNA was hybridised overnight to a HumanHT-12 v4 Expression BeadChip (Cat no. BD-103-0204, Illumina Inc., CA, USA), according to the Illumina protocol, in the Core Facility of the Department of Biotechnology of the University of Tartu. Samples were assigned to the arrays in a randomised order. The chips were scanned using Beadscan (Illumina Inc.).

### Methylation array

Genome-wide DNA methylation profiling was performed at the Institute for Molecular Medicine Finland (FIMM) Technology Centre of the University of Helsinki in two batches (time interval, 6 months). Bisulfite conversion of 1 μg of DNA was performed using the EZ-96 DNA Methylation Kit (Zymo Research, Irvine, CA, USA) according to manufacturer’s instructions. A 4-μl aliquot of bisulphite-converted DNA was subjected to whole-genome amplification and then enzymatically fragmented and hybridised to the Infinium HumanMethylation450 BeadChip (Illumina, San Diego, CA, USA) according to manufacturer’s protocol. Samples were assigned to the arrays in a randomised order. The BeadChips were scanned with the iScan reader (Illumina).

### Preprocessing of the methylation microarray data

The methylation data were preprocessed as a *methylumiset* object using R software with the *wateRmelon* array-specific package from Bioconductor [57]. The annotation information was based on the GRCh37/hg19 genome assembly from February 2009. Prior to any processing, all unspecific or polymorphic sites (n = 76775) were removed based on database information [58]. Samples and target sites of a technically poor quality were filtered out by excluding sites with a beadcount of <3 in 5% of the samples (n = 526) and sites for which 1% of the samples showed a detection p-value >0.05 (n = 740). Background correction and quantile normalisation via the *dasen* method were conducted individually for the two applied chemistries (Infinium I and II) as well as for the intensities of methylation (m) and un-methylation (u). After *dasen* treatment, the u and m intensities were transformed to beta (β) and M values. β is the ratio of the methylated probe (m) intensities to the overall intensities (m + u + α), where α is the constant offset, 100. Thus, β ranges linearly from 0 (non-methylated, 0%) to 1 (completely methylated, 100%). The β values were further transformed into M values using the equation log2(β/(1- β)). Next, the batch effect of the chemistries was adjusted using the BMIQ method, which is based on beta mixture models and the EM algorithm [59]. Several visualisation styles were used to verify the quality of the preprocessed data, such as boxplots from the raw intensities, Kernel density plots in the chemistry correction procedure and PCA plots (see Additional file 13). The batch effect of two laboratory days (time interval of 6 months) was confirmed via PCA (PC2 6.8%) to be a cause of severe bias in the data. Thus, the bias was corrected using an algorithm based on Empirical Bayes methods, as implemented in the R package *Combat* [60].

### Preprocessing of the gene expression microarray data

The gene expression microarray data were preprocessed as a *Lumibatch* object with the *lumi* pipeline using R software [61]. Background correction was performed with the *bgAdjust.affy* package. The gene expression values were then transformed with vst and normalised using the *rsn* method. Transcripts with transformed expression values of greater than 7.5 in 20% of the samples were included in the analysis. Visualisations, boxplots and PCA plots were used in the pipeline to verify the quality of the data.

### Comparison of age groups

To detect CpG sites showing substantial differences in DNA methylation between nonagenarians and young adults, the sites displaying the largest difference in the absolute value of the methylation level were included in the analysis (-1 > ∆M > 1, threshold for ΔM based on [61]). The rank-sums of the methylation values of the two groups were further compared with the Wilcoxon rank-sum test, and the nominal Benjamini-Hochberg-adjusted p-value was set to 0.05.

### Multiple regression analyses

To assess the relationship between age- and site-specific methylation levels in greater detail, a generalised regression model referred to as variable dispersion beta regression was utilised in an iterative manner (n = 407 646). Age group was employed as a predictor of the site-specific methylation outcome, in the form of β values (ranging from 0 to 1), in each equation of the mean model with a linker function of *logit*. Furthermore, as it was observed through PCA that the DNA methylation levels fluctuated based on the composition of blood cell subtypes, the proportions of CD28-/CD4+ and CD28-/CD8+ cells showed especially clear correlations with principal component 1, which explained 20% of the overall variance in DNA methylation. Therefore, the variables corresponding to cell type proportions (the CD4+ to CD8+ ratio and the proportions of CD28-/CD4+, CD28-/CD8+ and CD14+ cells) were set as adjustments in the analysis to determine leukocyte proportions independent of genome-wide ageing-associated DNA methylation changes. Sex was used as an additional covariate. The regression analyses were performed using R software and with algorithms implemented in the *betareg* package [62,63]. The nominal Bonferroni-adjusted p-value was set to 0.05. See Additional file 14 for a flow chart summary of the analysis steps to identify the high-confidence ageing-associated CpG sites.

### Correlations with gene expression levels

The associations between gene expression and DNA methylation levels were separately examined through bivariate correlation (Pearson) analyses for young and old individuals. The correlation analyses were designed for each transcript and CpG site pair showing identical annotation for a gene. Thus, multiple CpG sites were paired with the same gene, and several genes were matched with the same CpG site. In total, 2461 expression-methylation pairs were tested. The nominal Benjamini-Hochberg-adjusted p-value was set to 0.05.

### Pathway analyses

All the pathway analyses were performed on differentially methylated genes, i.e., genes that harbour at least one ageing-associated CpG site. There were 1832 hypermethylation-associated genes (3925 CpG sites) and 2057 hypomethylation-associated genes (4615 CpG sites) included in the dataset. Of the hypomethylated CpG sites, 1719 were not associated with any known gene, and of the hypermethylated CpG sites, 720 were not associated with any known gene.

IPA [34] was used to identify canonical pathways associated with our differentially methylated genes. According to the manufacturer, these canonical pathways are well-characterised metabolic and cell signalling pathways that have been curated and hand-drawn by PhD-level scientists. All the data sources provided by the Ingenuity Knowledge Base were included in the IPA, and the Ingenuity Knowledge Base was used as the reference set in all analyses. For the association of molecules, only experimentally observed results were accepted, and only human data were considered. Benjamini-Hochberg multiple testing correction (FDR) was employed to calculate the p-values for the pathways. Canonical pathways were considered significant at p < 0.05 (-logP > 1.3) and when the pathway contained a minimum of 3 genes. Pathways associated with cancer and other disease, as defined by Ingenuity Systems®, were excluded from the analysis. The IPA for hyper- and hypomethylation-associated genes was performed on 14.3.2014, and the IPA for genes showing a correlation between methylation and expression levels was performed on 12.3.2014.

GOrilla [31,32] was used to identify the enriched GO terms for the hyper- and hypomethylation-associated genes and for genes showing a correlation between the levels of methylation and expression. GO terms were searched based on two unranked lists (target and background), and all genes with at least one probe in the 450K array were used as the background list. A Bonferroni-corrected p-value of <0.05 was used as the threshold for significance.

PScan [33] can be used to predict if a group of genes is regulated by a common transcription factor. The analysis was performed with the default settings, i.e., using the Jaspar database and the -450 - +50 bp region around the TSS. PScan was able to identify 1811 and 2020 of the total hyper- and hypomethylation-associated transcripts, respectively. This analysis was performed on 11.3.2014. A Bonferroni-corrected p-value of <0.05 was used as a threshold for significance.

### *Array data*

The array data are available in the GEO database (http://www.ncbi.nlm.nih.gov/geo/) under the accession numbers GSE40366 for the gene expression data and GSE58888 for methylation data.

## Additional files

Additional file 1:
**All 8540 ageing-associated CpG sites.**
Additional file 2:
**Distribution of ageing-associated methylation sites across chromosomes.**
Additional file 3:
**Enriched GO function terms.** A table of GO function terms associated with identified ageing-associated methylation sites. GO function terms associated with hypermethylated sites in sheet a and GO function terms associated with hypomethylation in sheet b. Additional file 4:
**Diagram of enriched GO function terms.** A visualisation of Additional file 3. Additional file 5:
**Enriched GO process terms.** A table of GO process terms associated with identified ageing-associated methylation sites. GO process terms associated with hypermethylated sites in sheet a and GO process terms associated with hypomethylation in sheet b. Additional file 6:
**Diagram of enriched GO process terms.** A visualisation of Additional file 5. Additional file 7:
**Transcription factors predicted to regulate hypermethylation-associated genes.**
Additional file 8:
**Ingenuity canonical pathways associated with differentially methylated genes.** Canonical pathways associated with hypermethylation are in sheet a and canonical pathways associated with hypomethylation are in sheet b. Additional file 9:
**Sex- and age-group -associated methylation sites.**
Additional file 10:
**CpG sites showing a correlation to the expression level of the corresponding gene.**
Additional file 11:
**Genomic locations of the CpG sites where the level of DNA methylation correlates with the expression level of the corresponding gene.**
Additional file 12:
**GO process terms associated with genes whose expression levels were correlating with the level of DNA methylation.**
Additional file 13:
**PCA plots for quality control of methylation data.**
Additional file 14:
**Flow chart of the analysis steps used to identify ageing-associated methylation sites.**

## Abbreviations

27K array: Infinium HumanMethylation27 BeadChip
450K array: Infinium HumanMethylation450 BeadChip
Betareg: Beta regression model
ERV: Endogenous retrovirus
Non-CGI: Non-CpG island
PBMC: Peripheral blood mononuclear cell
TF: Transcription factor
TSS: Transcription start site
UTR: Untranslated region
