# Supplementary material for: Ageing-associated changes in the human DNA methylome: genomic locations and effects on gene expression
Source: BMC Genomics. 2015 Mar 14;16(1):179. doi: 10.1186/s12864-015-1381-z (PMC4404609; doi:10.1186/s12864-015-1381-z)
Supplement: Additional file 2: — Distribution of ageing-associated methylation sites across chromosomes. [file 12864_2015_1381_MOESM2_ESM.pdf]

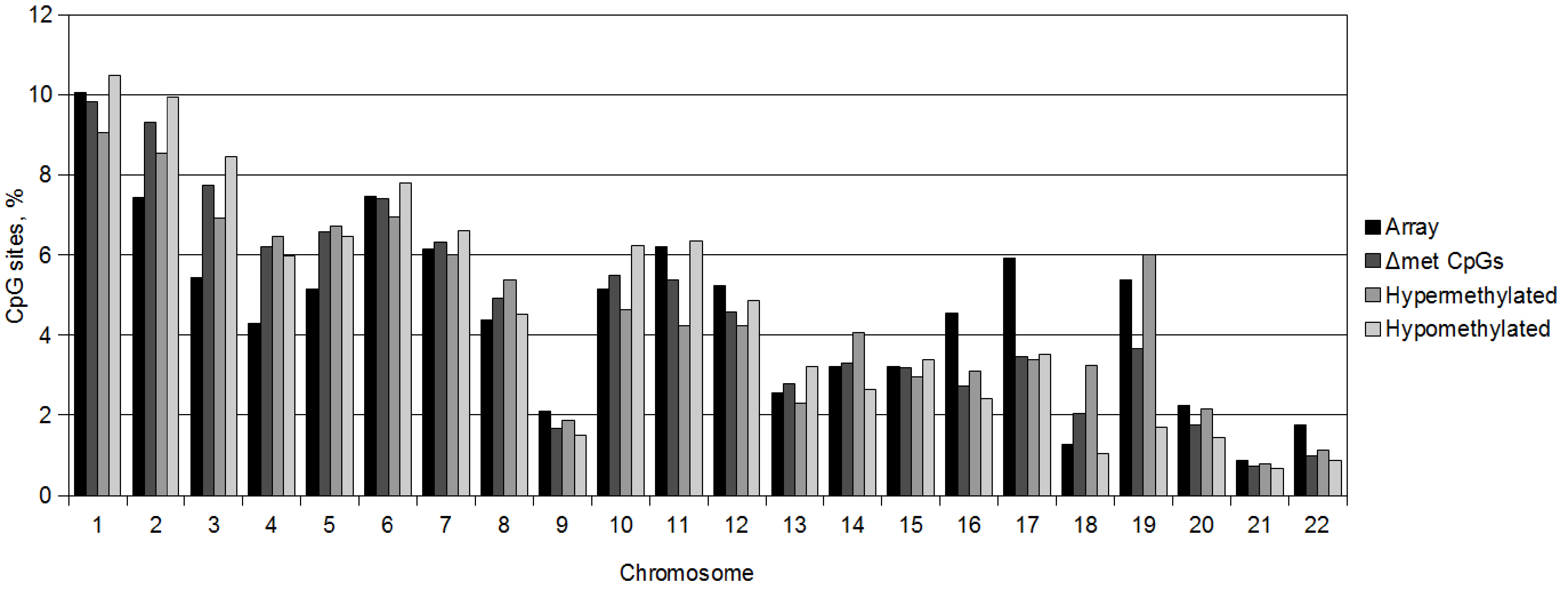

Additional file 2. The proportions of differentially methylated CpG sites across chromosomes. Array denotes the distribution of probes across the chromosomes, Δmet CpGs denotes the distribution of all 8540 differentially methylated CpG sites across the chromosomes and the terms hyper- and hypomethylated denote the distribution of ageing-associated hyper- and hypomethylated CpG sites across the chromosomes. On chromosomes 2, 3, 4, 5 and 18 we observed more differentially methylated sites than expected, whereas on chromosomes 16, 17, 19 and 22, we observed fewer differentially methylated CpG sites than expected. On majority of the chromosomes the proportions of hyper- and hypomethylated sites were as expected, except for chromosomes 18 and 19, where a clear over-representation of hypermethylated CpGs was identified.
