# Supplementary figures and images for: Ageing-associated changes in the human DNA methylome: genomic locations and effects on gene expression
Source: BMC Genomics. 2015 Mar 14;16(1):179. doi: 10.1186/s12864-015-1381-z (PMC4404609; doi:10.1186/s12864-015-1381-z)

## Hypomethylation associated GO function terms

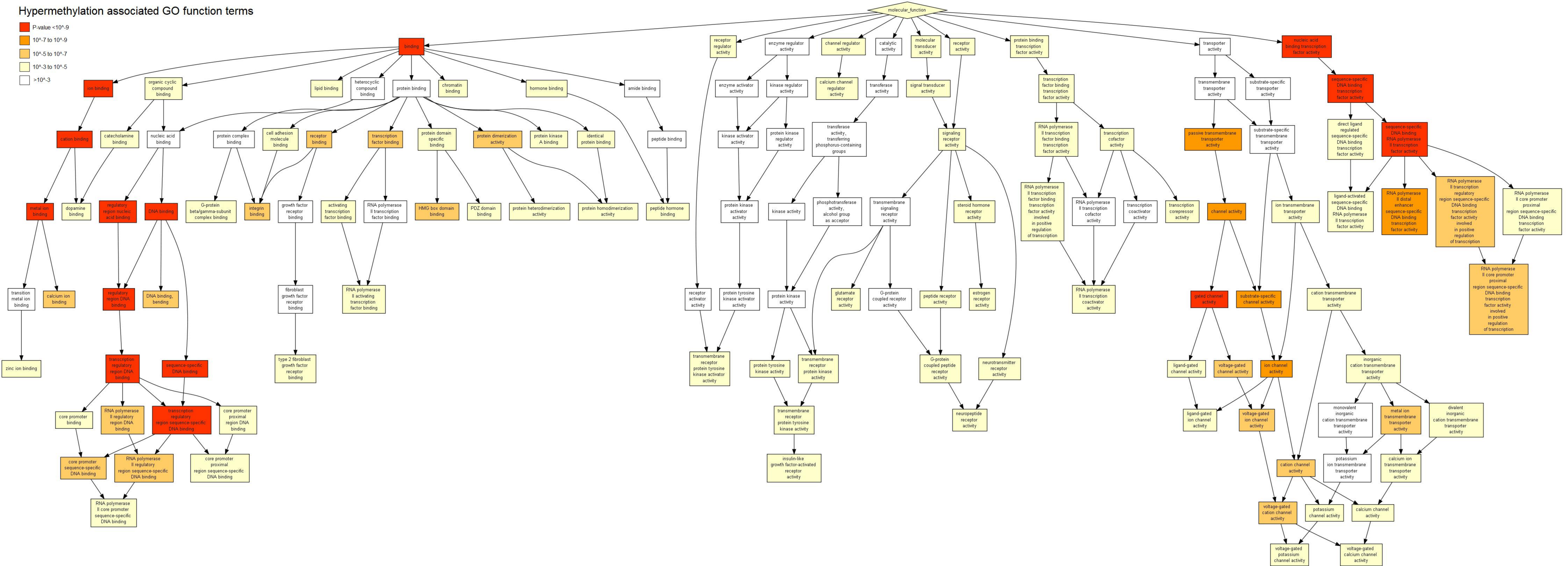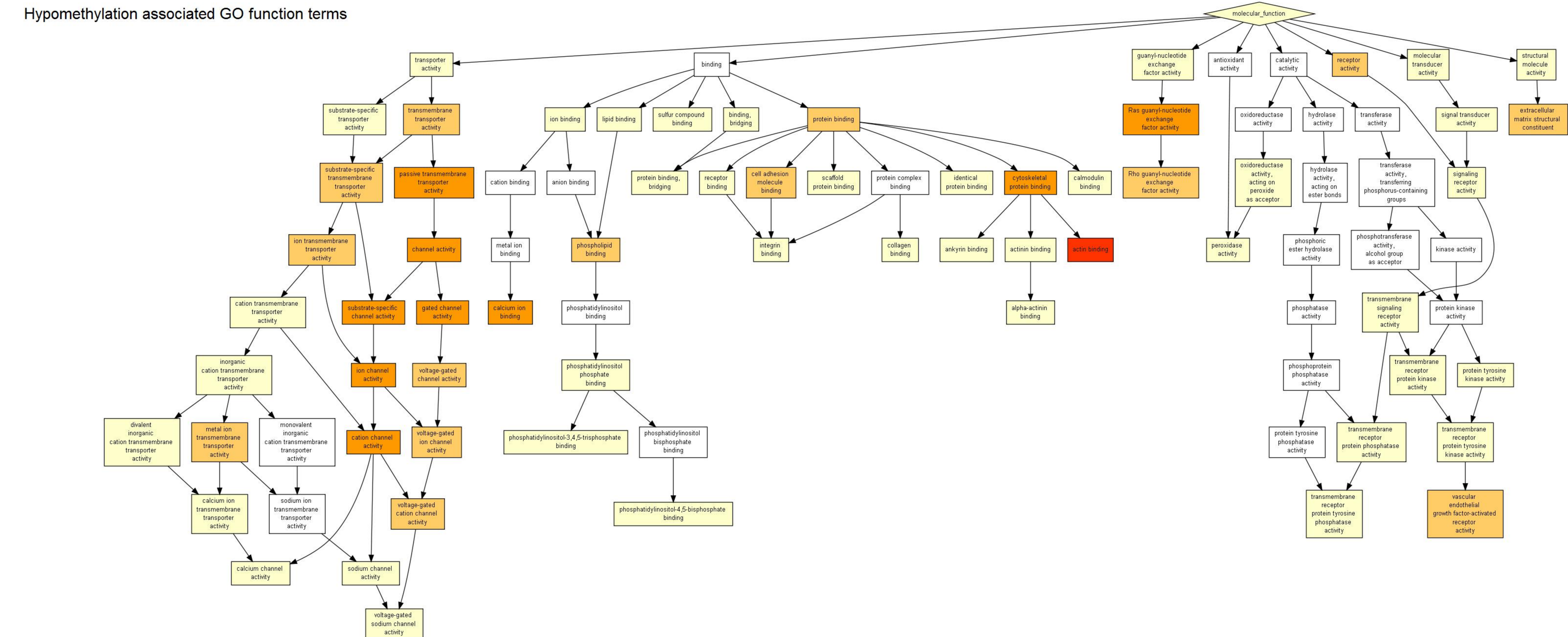

Supplement: Additional file 4: — Diagram of enriched GO function terms. A visualisation of Additional file 3. [file 12864_2015_1381_MOESM4_ESM.pdf]
