# Supplementary material for: Ageing-associated changes in the human DNA methylome: genomic locations and effects on gene expression
Source: BMC Genomics. 2015 Mar 14;16(1):179. doi: 10.1186/s12864-015-1381-z (PMC4404609; doi:10.1186/s12864-015-1381-z)
Supplement: Additional file 11: — Genomic locations of the CpG sites where the level of DNA methylation correlates with the expression level of the corresponding gene. [file 12864_2015_1381_MOESM11_ESM.pdf]

(a)

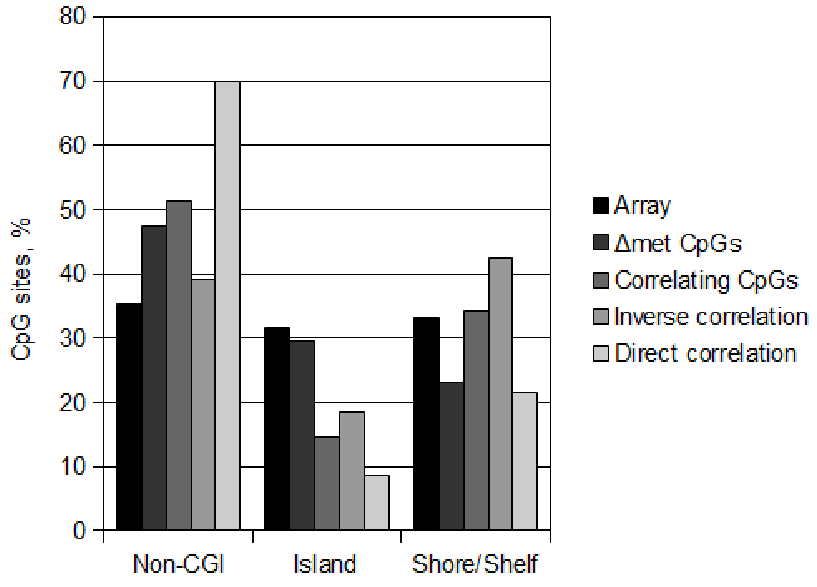

(b)

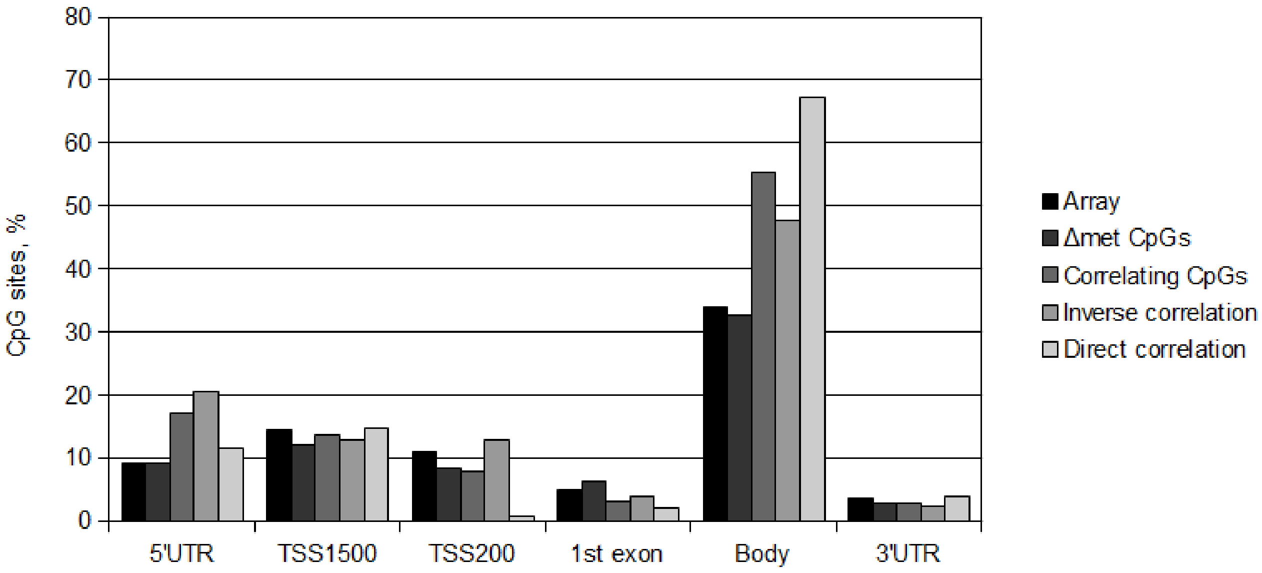

Additional file 11. Proportions of CpG sites with a correlation between the level of methylation and gene expression across the genome in relation to (a) CpG islands and (b) genes. Array denotes the distribution of probes in the array, Δmet CpGs denotes the distribution of the identified differentially with ageing methylated 8540 CpG sites and Inverse and Direct correlation denote the CpG sites where the level of methylation is inversely or directly correlated with the expression of the corresponding gene.
