# Supplementary material for: Ageing-associated changes in the human DNA methylome: genomic locations and effects on gene expression
Source: BMC Genomics. 2015 Mar 14;16(1):179. doi: 10.1186/s12864-015-1381-z (PMC4404609; doi:10.1186/s12864-015-1381-z)

Additional file 13. Paired plots from the principal component analysis for the DNA methylation data in the preprocessing stage. Panels present principal components (PCs) 1-3 with the largest proportion of explained variance before (panel a, upper) and after (panel b, lower) 'Combat correction'. The data is coloured based on the age group (young controls=black, nonagenarians=grey). PC2 clusters the data according to separate measurement days ('the batch effect') in panel a.

a)

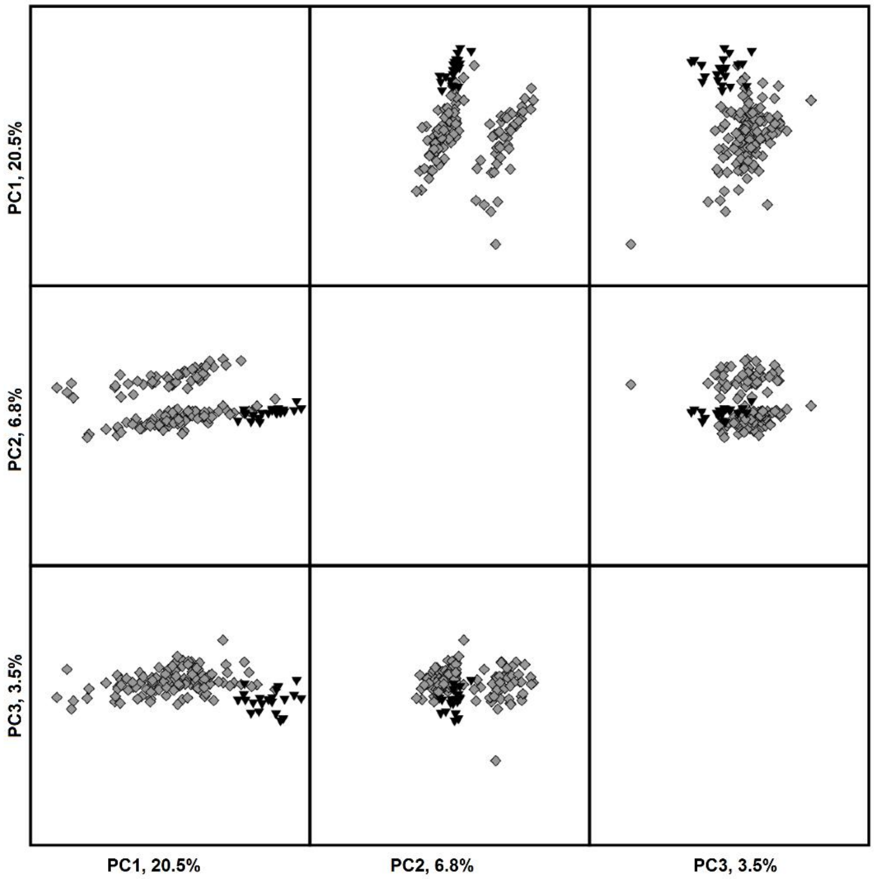

b)

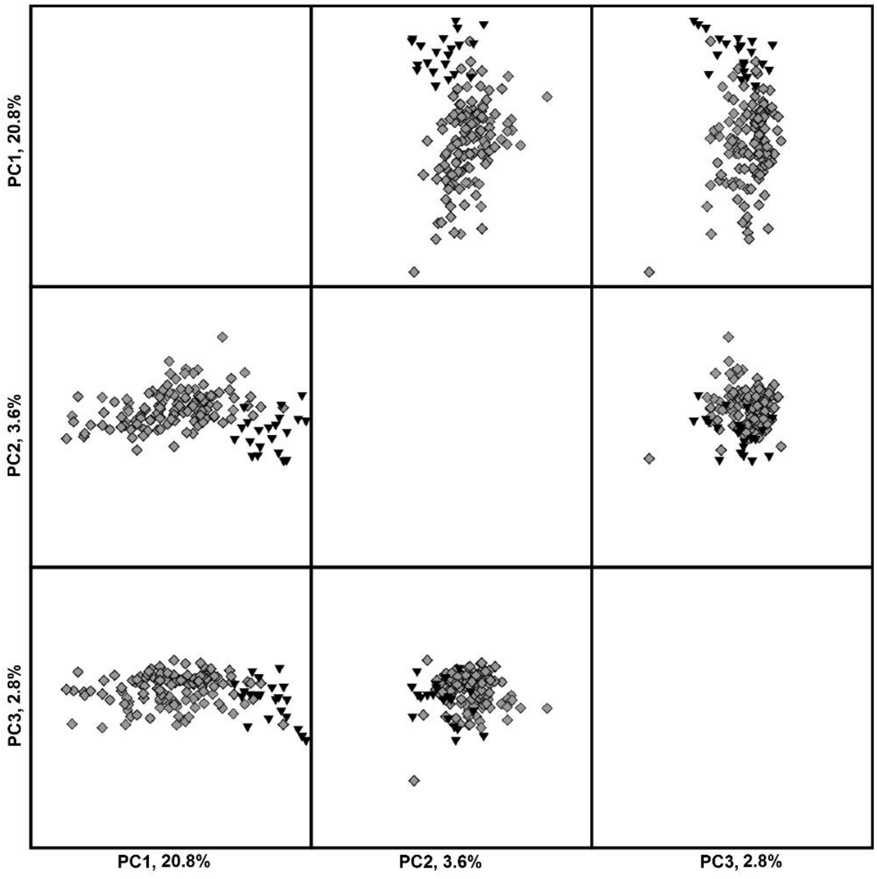

Supplement: Additional file 13: — PCA plots for quality control of methylation data. [file 12864_2015_1381_MOESM13_ESM.pdf]
