# Supplementary material for: Ageing-associated changes in the human DNA methylome: genomic locations and effects on gene expression
Source: BMC Genomics. 2015 Mar 14;16(1):179. doi: 10.1186/s12864-015-1381-z (PMC4404609; doi:10.1186/s12864-015-1381-z)
Supplement: Additional file 6: — Diagram of enriched GO process terms. A visualisation of Additional file 5. [file 12864_2015_1381_MOESM6_ESM.pdf]

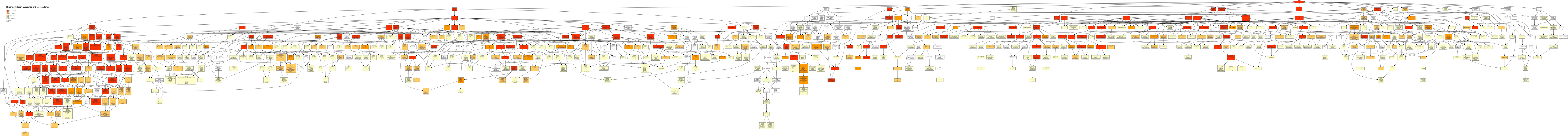
Hypermethylation associated GO process terms
P-value <10^-9
10^-7 to 10^-9
10^-5 to 10^-7
10^-3 to 10^-5
>10^-3

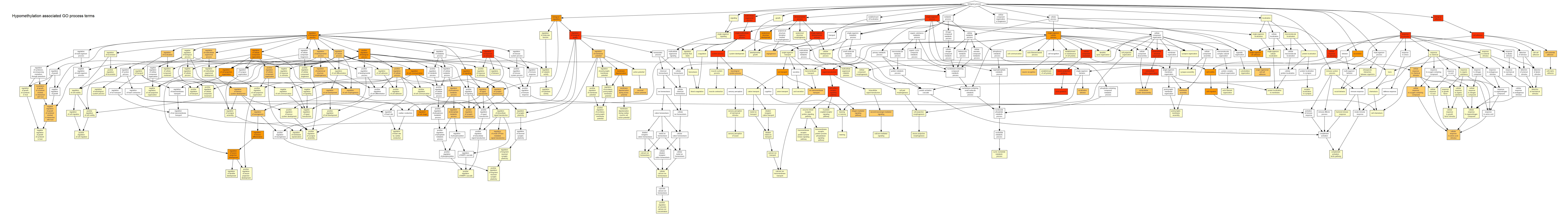
Hypomethylation associated GO process terms
