# Supplementary material for: Ageing-associated changes in the human DNA methylome: genomic locations and effects on gene expression
Source: BMC Genomics. 2015 Mar 14;16(1):179. doi: 10.1186/s12864-015-1381-z (PMC4404609; doi:10.1186/s12864-015-1381-z)
Supplement: Additional file 14: — Flow chart of the analysis steps used to identify ageing-associated methylation sites. [file 12864_2015_1381_MOESM14_ESM.pdf]

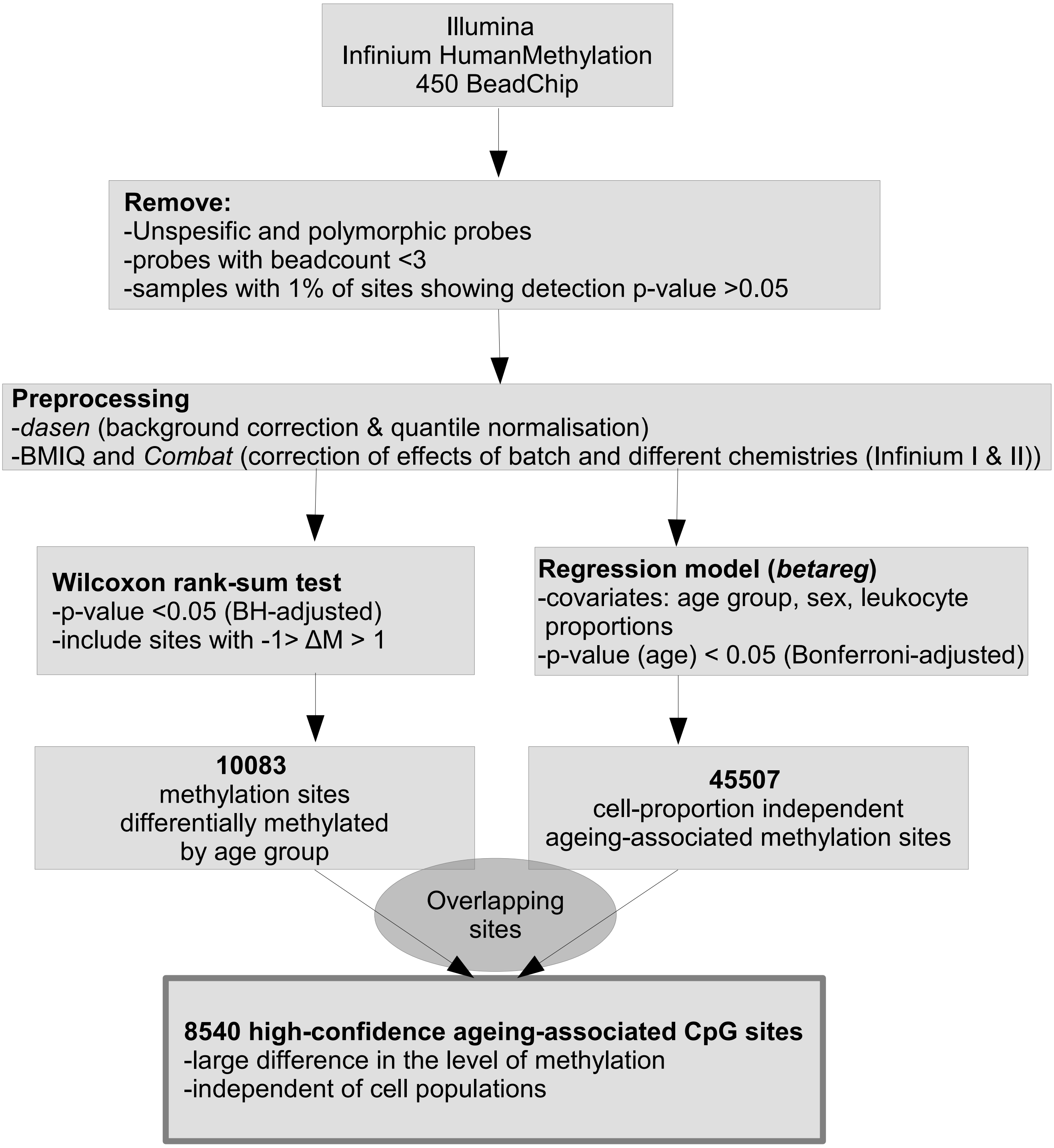

Additional file 14. A flowchart depicting the preprocessing and analysis steps to establish the set of ageing-associated probes. Wilcoxon rank-sum test was used to identify CpG sites with the largest difference in the methylation level between the age groups and the regression model was used to identify CpG sites whose level of methylation is associated with the age group independently of the changes in leukocyte proportions. CpG sites identified with both statistical methods are reported as high-confidence ageing-associated methylation sites. The great majority of the sites identified with both methods were the top hits of these methods, i.e. the sites had the largest difference in methylation level and a small p-value in the regression model.
